# Editor of New-York Journal of Medicine

**Published:** 1847-04

**Authors:** 


*Editor of New-York Journal of Medicine.*—It is gratifying to cite illustrations showing that mutual jealousies do not characterise, *par excellence*, as some suppose, members of the medical profession.—The following tribute to the talents and acquirements of the learned Editor of the New-York Journal of Medicine and the collateral Sciences, is from the Annalist, a spirited and able Journal, also published at New-York, and edited by Dr. Wm. C. Roberts. We feel that we do not incur the charge of invidiousness when we say that the encomiums are as worthily bestowed, as they are honorable to the writer:

"We are happy to acknowledge in this place his indefatigable zeal and great ability in the joint capacity which he exercises, of author and editor, and his high place among the learned in the profession, who do honor to it and to their country. His junior brethren are now reaping abundantly the fruits of his long and laborious study, which we hope he may be long spared to impart; and we trust that the journal over which he so successfully presides, may flourish, not less as a memento of the lamented genius who originated it, and whose premature loss Science, with unabated grief, continues to deplore, than to reward his own industry, and to increase his fame."

